# Risk Factors for Positivity to Shiga Toxin-Producing *Escherichia coli* and *Salmonella enterica* in Backyard Production Systems Animals from Metropolitana Region, Chile: A Threat to Public Health?

**DOI:** 10.3390/ijerph182010730

**Published:** 2021-10-13

**Authors:** Erika Pavez-Muñoz, Bastián Fernández-Sanhueza, Constanza Urzúa-Encina, Nicolás Galarce, Raúl Alegría-Morán

**Affiliations:** 1 Departamento de Medicina Preventiva Animal, Facultad de Ciencias Veterinarias y Pecuarias, Universidad de Chile, Santiago 8820808, Chile; isabelpm95@gmail.com (E.P.-M.); bastian.fernandez.s@ug.uchile.cl (B.F.-S.); constanza.urzua23@gmail.com (C.U.-E.); ngalarce@ug.uchile.cl (N.G.); 2 Central Veterinary Research Laboratory, Facultad de Ciencias Veterinarias y Pecuarias, Universidad de Chile, Santiago 8820808, Chile; 3 Facultad de Ciencias Agropecuarias y Ambientales, Universidad del Alba, Santiago 8370007, Chile

**Keywords:** backyard production systems, STEC, *Salmonella* *enterica*, risk factors, public health, One Health, zoonosis

## Abstract

In the Metropolitana region of Chile there are 3836 backyard production systems (BPS), characterized as small-scale systems. They act as a source of zoonotic pathogens, such as *Salmonella enterica* and Shiga toxin-producing *Escherichia coli* (STEC), whose prevalence in BPS has not been fully described. The objective of this study was to determine the positivity for both agents in BPS and to establish the risk factors related to their presence. In each BPS, an epidemiological survey was undertaken, and stool samples were collected to detect these pathogens via bacteriological culture and conventional PCR techniques. Subsequently, multivariable logistic regression models were applied to establish the risk factors associated with their presence. BPS positivity rates of 11.76% for STEC and 4.7% for *S. enterica* were observed. The systems showed poor welfare standards and a lack of biosecurity measures. The risk factor analysis concluded that the Gini–Simpson index (*p* = 0.030; OR = 1.717) and the presence of neighboring intensive poultry or swine production systems (*p* = 0.019; OR = 20.645) act as factors that increased the risk of positivity with respect to STEC. In the case of *S. enterica*, exchanging embryonated eggs (*p* = 0.021; OR = 39) and the presence of debeaked chickens (*p* = 0.001; OR = 156) were determined as factors that increased the risk of positivity for this agent. For positivity with respect to both pathogens, the Gini–Simpson index (*p* = 0.030; OR = 1.544) and being INDAP/PRODESAL users (*p* = 0.023; OR = 15.026) were determined as factors that increased the risk, whereas the type of confinement (*p* = 0.002; OR = 0.019) decreased it. Epidemiological surveillance of these neglected populations is lacking, highlighting the fact that STEC and *S. enterica* maintenance on BPS represents a potential threat to public health.

## 1. Introduction

### 1.1. Backyard Production Systems

Backyard production systems (BPS) are considered one of the most common forms of animal production in the world, especially in developing countries [1]. BPS are small home-based production systems with low technological development, located mainly in rural areas [2,3,4]. Although they are small-scale systems, BPS constitute part of the household income through the domestic consumption of animal protein and subproducts and the informal sale of organic farm products; a market that has gained popularity in the last few years as a result of the new food preferences of consumers [5,6]. In this regard, BPS have been found to be socioeconomically valuable in various rural communities around the globe, including Chile [7,8,9].

One of the main characteristics of BPS is their lack of biosecurity measures [7,10] and their limited use of veterinary assistance, which may limit the early detection of infectious diseases caused by zoonotic pathogens [4]. Additionally, a high rate of contact between the different animal species kept in BPS can be observed [6], leading to the maintenance and dissemination of a wide range of pathogens [11,12,13]. Among these are enteric bacteria such as STEC and *S. enterica*; zoonotic pathogens considered as two of the main causes of foodborne illness globally [14,15,16,17]. A total of 3 million acute cases, 3890 cases of hemolytic uremic syndrome (HUS) and 230 deaths per year worldwide are estimated to be due to STEC [18]. For non-typhoidal salmonellosis, around 535.000 acute cases and 77.500 deaths are estimated to have occurred in 2017 [19].

### 1.2. Shiga Toxin-Producing E. coli and Salmonella enterica

Both *E. coli* and *S. enterica* are Gram-negative, facultative anaerobic bacilli; members of the Enterobacteriaceae family, which are the most abundant enteric commensal and pathogenic agents in homeothermic animals [20,21]. *E. coli* can be classified in eight pathotypes, with STEC being one of the zoonotic pathotypes [22,23]. The characterization of STEC is based on the detection of the genes encoding for Shiga toxins (Stx): Stx1 and Stx2 with their respective subtypes [24]. In the case of *S. enterica*, more than 2650 serotypes of *S. enterica* have been described [25]. 

Both pathogens can cause asymptomatic infections or self-limiting gastrointestinal signs [26,27], but they can also cause a severe clinical presentation, extraintestinal disease or even death in the at-risk population, including immunocompromised patients, children, pregnant women and seniors [27,28]. STEC is mainly associated with diarrhea, which can be hemorrhagic. In addition, it can produce HUS in the at-risk population [21], which can be life-threatening or can lead to renal, gastrointestinal, cardiovascular or central nervous system complications, especially in children [29]. *S. enterica* normally causes self-limiting diarrhea, but can also generate severe disease and can progress to bacteremia with extraintestinal complications [27], including pneumonia, cholecystitis, cellulitis, pancreatitis, urinary tract infections, appendicitis, endocarditis and meningitis [30]. 

The horizontal route is the most important for the transmission of both agents. Transmission via the oral–fecal route or consumption of water or food contaminated with waste or feces, represent the main sources of infection for humans [30,31]. Both pathogens are frequently isolated in cases of foodborne diseases [14,15,16,17], with detection mainly on poultry carcasses, eggs, dairy products and beef [32].

The role of several animal species as a reservoir for these pathogens has been previously described, including wild and domestic species such as rodents, reptiles, poultry and wild birds, dogs, cats and pigs [32,33]. Cattle are recognized as one of the largest reservoirs of STEC [21], whereas hens and swine are the most important reservoirs of *S. enterica* [34]. Animal reservoirs are generally asymptomatic, although young animals may become ill [29]. BPS animals can contaminate food or watercourses, infecting other animals and potentially transmitting these pathogens to humans [30,35].

Worldwide, and particularly in Latin America, the *S. enterica* and STEC prevalence in BPS is largely unknown. In cattle, the prevalence of STEC O157 is reported to range from 0 to 71%, and in some herds the infection rate is as high as 100% [36]. The prevalence has been described as ranging between 0.2 and 74% in dairy cattle [36,37], as over 70% in sheep and goats [38] and as 4% in captive wild birds [39], with several other reports of its presence in other animal species that have close contact with humans [40,41]. In contrast, the prevalence of *S. enterica* at the BPS level is reported to range from 3.5% to 31.0% in hens reared under these low-tech production systems [42,43], demonstrating that these animals are not part of any surveillance system in the region.

In animals, limited risk factors have been described for STEC, including low levels of technification. Calf groups have been reported as factors that increase the prevalence in cattle [44]. Risk factors for positivity with respect to *S. enterica* in BPS have been reported only to a limited extent, due to major gaps in knowledge regarding this high-risk population. Larger flocks of chickens, the number of other bird species (e.g., geese, turkeys or ducks), free-range birds, BPS mixed production and mixing replacement animals all lead to a significantly higher risk of positivity with respect to *Salmonella*. Implementing any action when the disease was present led to a significantly lower risk of becoming *Salmonella*-positive [6,42].

### 1.3. Chilean Situation

Information on the sanitary status of both bacteria in BPS is scarce [1], partly because their clinical presentation is mostly asymptomatic in animals [45]. The prevalence of *S. enterica* has been reported to be between 4.2 and 21.23% in hens and swine bred under BPS in central Chile [6,46,47]. For STEC, a recent study reported positivity rates of 17% in cattle and 1% in pigs, where both species were from commercial farms [48]. In addition, the following factors are described as factors that increase the risk of positivity for *S. enterica* in BPS: bird species diversity (odds ratio (OR) = 1.04, 95% CI: 1.01–1.07), BPS with mixed production activities (agricultural and livestock) (OR = 5.35, 95% CI: 1.2–27.6) and BPS that obtained replacement animals from various sources (OR = 5.19, 95% CI: 1.4–20.5) compared to those using their own animal replacements [6].

No information has been reported in relation to the prevalence and risk factors for STEC in BPS in Chile to date. For *S. enterica*, even when national evidence has been reported, the information is limited in terms of risk-factor determination. This highlights the great gap in the information available regarding both bacteria in BPS animals, which could lead to an increase in the risk of transmission, presenting a threat for animal and/or public health.

Based on all the above, it is necessary to increase the existing information regarding these two bacteria and their presence and behavior in BPS. The aim of this study was to determine the risk factors associated with positivity for both bacteria in terms of management, biosecurity aspects, sanitary status and relationship with the environment, by characterizing these production systems and establishing their positivity rates for *S. enterica* and STEC, assuming the circulation of these two important bacteria among animals raised in BPS.

## 2. Materials and Methods

### 2.1. Study Design and Sample Unit Determination

Stratified and proportional random sampling was performed, based on the provinces within the Metropolitana region (Chacabuco, Cordillera, Maipo, Melipilla, Santiago and Talagante). The sample size was calculated using the following equation [49]:

*n* = *Z*_*α*_^2^*pq*/*L*^2^ (1)

where *n* represents sample size and *Z_α_* is the required value for confidence = 1 − *α*, where α corresponds to the confidence level set at 95%, *Z_α_* is the (1 − *α*/2) percentile of a standard normal distribution, *p* is the expected prevalence of the pathogen, *q* = 1 − *p* and *L* is the precision of the estimation or margin of error set at 5%. Based on previous studies of the epidemiology of *S. enterica* in BPS in the Metropolitana region, the sample size was calculated using a prevalence of 5% [6]. BPS that maintained at least one productive animal species (mainly poultry and swine) were selected. Based on the above and on the information from the last Animal and Forestry census carried out by the *Instituto Nacional de Estadísticas de Chile* (INE) [50], a sample size of 73 BPS was determined (Table 1).

The intra-BPS sample size was calculated using the following equation [49]:

*n* = (1 − *α*^1/*D*^)(*N* − (*D* − 1)/2) (2)

where *n* represents the sample size, *N* is the sample size of BPS, *D* is the minimum estimated number of sick animals in the group and α = 1 − the confidence level. Considering the detection of at least 30% of positive animals, a minimum sample size of 8 animals in each BPS was determined. In those cases where it was not possible to capture the minimum number of animals to sample, fresh environmental samples were collected.

### 2.2. Epidemiological Data Collection

During the field activities, a previously validated [6] survey was applied in each BPS (applied questionnaire in Supplementary Material), in order to characterize the factors that may influence the maintenance and dissemination of the pathogens under study, including handling, the presence or absence of specific biosecurity conditions, socioeconomic variables and animal exchange. The respondent’s names, as well as the georeferences, were codified and blinded for the research group. Only the PI had access to the information in full detail. The interviews were conducted in Spanish by the corresponding author of this manuscript, who has been trained in epidemiologic questionnaire application. None of the surveyed BPS owners had any relationship with any member of the research team and all survey respondents were over 18 years old. Each interview lasted an average of 20 minutes. Survey questions were aimed at poultry and swine breeding, since these are the most common species reported as being kept in BPS. The information was manually collected and then digitized, processed and managed using Microsoft Excel^®^ (version 16.50). Informed consent was obtained to ensure that both the information collected through the survey and the results of the analysis of the sample were treated confidentially, and to allow the publication of global project results while protecting personal data. The questionnaire was approved by the bioethics committee of Universidad de Chile, under the number 18205-VET-UCH. All BPS owners were informed about the results of the samples collected from their animals. 

### 2.3. STEC and S. enterica Positivity Establishment

Fecal samples were collected from animals raised in BPS, taken directly from the cloaca in birds or from the rectum in mammals, using sterile swabs with Cary–Blair transport medium (Copan^®^, Italy). Additionally, environmental samples were collected only under the following conditions: the presence of fresh feces and species identification. Samples were transported and stored at 4 °C until processing.

For STEC detection, protocols for bacteriological cultures described previously were followed [32,51]. The STEC culture protocol includes: pre-enrichment of the samples with 5 mL of trypticase soy broth (Becton, Dickinson and Company, Franklin Lakes, NJ, USA), incubated at 42 °C for 18 to 24 h, followed by enrichment and isolation of a homogeneous aliquot on MacConkey Agar (Becton, Dickinson and Company, Franklin Lakes, NJ, USA), incubated at 37 °C for 18 to 24 h. Those plates showing bacterial growth were confirmed using conventional PCR techniques. The presence of Stx1 and/or Stx2 genes was assessed via PCR with primer sets and reaction conditions following protocols previously described [51].

The protocol for *S. enterica* isolation consisted of three stages: pre-enrichment of samples with 5 mL of phosphate buffered peptone water broth (APT, Difco^®^), incubated at 37 °C for 24 h, followed by plate culture with modified semi-solid Rappaport–Vassiliadis medium (MSRV, Oxoid^®^), incubated at 42 °C for 24 to 48 h. Finally, those plates showing growth were isolated on plates with xylose lysine deoxycholate (XLD, Difco^®^) selective medium, incubated at 37 °C for 24 h. Plates with concentric black or red colonies, without color change, were confirmed using conventional PCR techniques, searching for the *inv*A gene according to previously described procedures [52].

### 2.4. Risk Factor Determination

Since the response variable (positivity to STEC and/or *S. enterica*) was dichotomous, where Y can take only two values, 0 or 1 (Y = 0 or Y = 1), representing the absence (0) or the presence (1) of the studied agent [49,53], three multivariable logistic regression models were built: one for STEC, one for *S. enterica* and one that grouped both pathogens (called the Enterobacteria model) [49,54,55].

For the selection of variables to be analyzed as potential risk factors, a univariable logistic regression analysis was performed for all the variables reported in the survey, and those with a *p*-value of less than 0.15 (liberal *p*-value criterion) were selected [49]. A cut-off value of *p* < 0.20 was also evaluated, showing no difference in the variable selection. All the variables that fulfilled this criterion were analyzed using Spearman’s correlation for quantitative variables and Fisher’s exact test for qualitative variables, to check the collinearity and the association between variables, allowing correction for potential confounding factors.

The multivariable logistic models were built using the following equation [49,53]:

(3) $$ln\left(p/1-p\right)=\beta_{0}+\sum \beta_{j}X_{j}\;$$

where *p* is the probability of the outcome at any value of *x*, *β*_0_ corresponds to the intercept and ∑*β_j_X_j_* is the summation of the effect (*β_j_*) of each independent variable (*X_j_*) added into the model [54].

The construction of the multivariable model was subjected to a stepwise backward elimination procedure, removing from the model those variables that presented non-significant regression coefficients (*p* > 0.05) when the models were compared using the likelihood ratio test (LRT) [54]. Those variables that were not significant for the construction of the model and which, when eliminated, modified the regression coefficients of the remaining variables by more than 20%, were retained in the final model, to adjust for confounding factors. The convergence of each model was set at epsilon (ε) = e^−16^, in order to present stricter conditions for determining statistically significant factors. The final model was the one that presented the lowest record in the LRT [54]. The goodness of fit was assessed using the Hosmer–Lemeshow test [54,56].

In order to have a variable that takes account of the diversity of species present in BPS as a potential risk factor, the Gini–Simpson index was calculated [57]. In addition, interactions in a biological and/or epidemiological sense were included between the evaluated variables.

All the analyses were performed using the statistics software R version 4.1.0 [58] and RStudio [59].

## 3. Results

### 3.1. Characterization of the Sampled BPS

A total of 85 BPS were sampled in the Metropolitana region. When broken down at the province level, a total of 34 BPS were sampled in Melipilla, 13 in Chacabuco, 10 in Santiago, 5 in Cordillera, 7 in Talagante and 16 in Maipo. An average of 8.38 samples were collected in each BPS. The epidemiological survey showed that, on average, families consisted of four or five members. When asked about their production systems, 51.76% (44/85) of owners reported having a mixed production, shared between agriculture, forestry and livestock. Regarding the occupations of BPS’ owners, 36.47% (31/85) declared themselves to be pensioners, 37.64% (32/85) agricultural workers and 25.88% (22/85) non-agricultural workers. Owners were also asked to categorize the importance of animal breeding for the household economy on a scale of 1 (not important) to 5 (very important). In general, each category presented relatively homogeneous frequencies (1 = 16/85, 2 = 12/85, 3 = 16/85, 4 = 25/85 and 5 = 16/85).

The BPS visited were composed of a great diversity of species, including domestic birds, large and small ruminants, other herbivores, swine and pets (Table 2). The median obtained for the total number of animals in each sampled BPS was 40 animals. From this data, the Gini–Simpson diversity index was calculated for each BPS.

In terms of BPS characterization, animal management was carried out mostly by women (36/85), followed by men (23/85) and families (22/85). Most BPS owners reported that they had kept and bred animals, with the aim of domestic consumption and to sell their products, for more than twenty years. The animals were generally kept under mixed confinement. Despite having functional fences, contact was possible between different species in the BPS and also with neighboring BPS animals, wildlife and visitors to the BPS. Replacement animals mainly originated from the same system or, to a lesser extent, from multiple sources including fairs and neighboring farms. Very few BPS owners claimed to exchange embryonated eggs or keep debeaked chickens, and none of them kept tail-docked swine. Most BPS did not receive veterinary assistance; the owners dealt with management and administering drugs or other treatments to sick animals themselves. In addition, relationships between BPS owners and governmental entities in charge of providing support to this type of system were scarce. Further information on BPS characterization is given in Table 3.

### 3.2. Positivity to STEC and S. enterica

Of all the BPS analyzed, ten were positive for STEC and four were positive for *S. enterica*, representing positivity rates of 11.76% and 4.71%, respectively (Table 4). With regard to to the species involved in STEC positivity, ruminants (cows and sheep) were the most common species found to be positive for this agent, followed by poultry (chickens and ducks) and pigs (Table 5). In the case of *S. enterica*, birds (chickens and geese) were the main group to show positivity for this agent. A total of 712 samples were collected, obtaining 20 (2.81% sample positivity) STEC-positive samples, with 80% positive for Stx1 (16/20) and 40% positive for Stx2 (8/20), together with 5 (0.70% sample positivity) *S. enterica*-positive samples (Table 5).

### 3.3. Risk Factors for STEC Positivity

Regarding the constructed epidemiological model, the variables that were shown to be statistically associated with positivity with respect to STEC were the Gini–Simpson index (OR = 1.717; CI-95%: 1.054–2.799; *p* = 0.030) and the presence of neighboring intensive poultry or swine production systems (OR = 20.645; CI-95%: 1.648–258.706; *p* = 0.019). Both acted as factors that increased the risk of positivity (Table 6).

### 3.4. Risk Factors for S. enterica Positivity

The significant variables in the epidemiological model for *S. enterica* positivity were the exchange of embryonated eggs, behaving as a factor that increased the risk of positivity (OR = 39; 95% CI: 1.745–871.724; *p* = 0.021) and the presence of debeaked chickens, also being a factor that increased the risk of positivity (OR = 156; 95% CI: 6.979–3486.896; *p* = 0.001) (Table 7).

### 3.5. Risk Factors for STEC and S. enterica Positivity

The model obtained for both bacteria had the following significant variables: the Gini–Simpson index, the type of confinement under which the animals were kept and whether the BPS was a user of the *Instituto de Desarrollo Agropecuario* (INDAP) or *Programa de Desarrollo Social* (PRODESAL). Both the Gini–Simpson index and whether the BPS was a user of INDAP or PRODESAL, were factors that increased the risk for positivity to both pathogens (OR = 1.544; 95% CI: 1.044–2.284; *p* = 0.030 and OR = 15.026; 95% CI: 1.465–154.082; *p* = 0.023, respectively). In contrast, the type of confinement was a factor that decreased the risk for positivity to the studied agents (OR = 0.019; 95% CI: 0.001–0.244; *p* = 0.002), in cases where the animals were kept under a mixed confinement system (Table 8).

## 4. Discussion

### 4.1. Economic Perspective of BPS in the Chilean Context

BPS are important for the community, because they organically produce foods of animal origin, which, according to current food trends, are preferred by consumers [5]. In general, the BPS characterization in this study reported similar results to those observed previously, both in Chile and in the rest of the world, highlighting a great diversity of intra-BPS species destined for domestic consumption or sale, kept using low biosecurity measures with little veterinary assistance, where such production was not the main economic activity of the household [3,4,6,7]. It should be noted that the importance of animal breeding for the household economy varied considerably between BPS. In this sense, animal production can be presented as the main or a secondary business, implying that at least a significant proportion of BPS owners must perform other jobs or activities in addition to animal breeding, to maintain the household economy. This situation limits the amount of time available for BPS management, thus increasing biosecurity deficits [60]. BPS and integrated production systems are key to promoting the local economy in undeveloped and developing countries [61].

### 4.2. BPS Management Characterization

Characterization showed that STEC- and *S. enterica*-positive BPS owners do not normally associate with, or receive veterinary advice from, official entities such as SAG, INDAP or PRODESAL (veterinary or agricultural advisory institutions belonging to the government (central and/or regional)), nor from private professionals (veterinarians or veterinary technicians). In addition, when a relationship between the BPS and the veterinary services exists, it is generally associated with swine breeding, due to the higher technical requirements for their maintenance and the existence of diseases that affect this species and are under official surveillance plans (e.g., PRRS, avian influenza and swine influenza). However, the number of BPS owners who report keeping pigs is low, which is reflected in the low rate of technical and health assistance that the surveyed BPS received. The absence of technical–sanitary assistance and the scarce implementation of biosecurity measures could increase the maintenance of zoonotic and non-zoonotic pathogens in BPS, with the consequent risk of contagion for the people in charge of these systems and the susceptible animal species kept in them or in neighboring systems (which could include small-scale and large-scale production systems) [62,63]. Only five BPS, of all those sampled, were visited by SAG more than once. This low frequency of visits could be an indicator of good health status, although, based on the observed handling and biosecurity characteristics, this situation is more likely to be explained by other factors. To make a more accurate estimate, it would be necessary for the BPS present in this region to be integrated transversally into the surveillance programs carried out by official entities [64,65,66,67].

Among the studied variables associated with biosecurity measures in BPS, the presence of a footbath and the practice of disinfection before entering and leaving the farm could not be included in the risk factor analysis, because the results did not show variability since there was no presence of the aforementioned protocols in any of the sampled BPS. This situation was corroborated by field inspection. This reinforces the hypothesis that BPS are themselves a promoting factor for the contact of zoonotic pathogens with the population. One example is the handling of animals while wearing clothes or footwear common to the rest of the daily activities, without rigorous disinfection or sanitation. These elements can then act as fomites, with the potential to infect other people or disseminate pathogens to other systems that may be visited by the BPS owners [60,68].

Another important characteristic to highlight is that, of the surveyed BPS, those in charge of maintaining them were mainly pensioners (older than 60 years); a situation that has been reported previously [7]. In Chile, this could be a factor that influences the lack of, or delay in, the adoption of new practices that could improve the sanitary status of these productive systems [69]. There is also a perception of mistrust in those in charge of BPS directed towards official veterinary and health authorities, which is reflected in the low participation rate of these entities in the visited BPS. An explanation for this may be that these organizations play an active role mainly against outbreaks of diseases of importance to commercial farms, such as avian influenza or PRRS, where the most common control measure involves the elimination of the affected animals [7,70]; an act that is not usually accompanied by financial compensation and therefore has an impact on the household economy [70].

Despite the fact that BPS owners reported infrequent use of pharmacological treatments, the administration of pharmaceutics to treat animals without a medical prescription was observed; an important situation since there is previous evidence of the presence of antibiotic residues in Chilean BPS [71]. Hence, the administration of pharmacological treatments could be underestimated, since at the time of sampling boxes and bottles of medicines such as antibiotics and analgesics were observed around pens. Additionally, there was a lack of knowledge about the concept of “vaccination”, with owners understanding it as the injection of any kind of drug or product. This fact may be an indirect indicator of a possible underestimation or overestimation of immunity levels for certain diseases [72,73]. The consequences of these practices could have an impact on public and animal health, mainly associated with the generation of antimicrobial resistance against widely used drugs [34,35,48,71,74,75]. Previous studies have detected multidrug resistance in non-typhoidal *Salmonella* strains from Chilean BPS [76] and sensitivity to a wide range of antimicrobials in STEC isolates from backyard animals [75].

### 4.3. STEC and S. enterica Positivity in BPS

Recent reports in Chile indicate STEC positivity rates of 17% in cattle and 1% in slaughtered pigs [48]. In addition, reports on meat, seafood, vegetables and ready-to-eat street-vended food samples indicate 0.5% positivity (18/3300) [77]. However, the results obtained for STEC in this study are the first reported at BPS level in Chile. This is important, given the potential risk to public health mainly associated with close contact between animals and people who are in charge of these systems, and the possible risk of occupational disease. Human–animal contact could be even higher than in commercial farms, where biosecurity measures are also stricter [68].

The positivity rates for *S. enterica* reported in this study are similar to those previously described for BPS in Chile [6,46] and in other countries [42,43,78,79]. In terms of the risk factor analysis, evidence suggests that those variables that were found to be significant and increased the risk of positivity with respect to *S. enterica* in this study were also found to be significant in previous studies [6].

The animal species that showed positivity to STEC and/or *S. enterica* were consistent with the reservoirs previously described in the literature for both pathogens [21,32,33,37].

### 4.4. Risk Factors Associated with STEC and S. enterica Presentation in BPS

In this study, two variables were found to be significantly associated with positivity to *S. enterica* in BPS: exchanging embryonated eggs and the presence of debeaked chickens, both increasing the risk of positivity to this pathogen. This situation could be explained because these two variables are a reflection of the potential relationship between BPS and commercial flocks [80]. Debeaking is a common and recommended practice in intensive systems to avoid behaviors such as cannibalism and pecking during the production process [81], consequently it is a common practice in large-scale production systems. The presence of debeaked chickens could provide evidence of the existence of animal movement, with the possible acquisition of replacement animals from commercial flocks in BPS and the selling of embryonated eggs, contributing to the dissemination of pathogens and their circulation [34]. It should be mentioned that the prevalence of *S. enterica* and STEC in animals from commercial production systems is usually higher than in BPS [48,82,83,84]. As additional background, both factors were found at a low frequency within the sample, which could indicate that relationships between commercial production systems and BPS are infrequent.

The Gini–Simpson index of species diversity was shown to be a statistically significant variable in both the STEC and the Enterobacteria models. This indicates that, since a BPS has more species (giving an index value closer to 1), there is a greater risk of these systems showing positivity to STEC or to both studied agents at the same time. The existence of more than one species on a farm could be an indicator of a high rate of contact between different animals, promoting the appearance of emerging diseases and their spread to other BPS [68]. Additionally, for the STEC model, the presence of nearby commercial production systems also acted as a variable that increased the risk of positivity. This could be related to the aforementioned points regarding the contact of BPS with commercial plants, considering that the latter tend to present higher prevalence values for several pathogens of importance to public and animal health [48,82,83,84]. These results highlight the importance of surveillance in this neglected animal and human population, as evidence suggests that direct contact with farm animals could act as a predictor of progression to HUS in human cases of STEC infection [85,86].

Finally, the type of confinement in which the animals are kept was determined as a factor that decreased the risk of positivity to both of the enterobacteria under study. Animals kept in a mixed confinement system might have a lower rate of contact with other animals and people, thus decreasing the probability of pathogen dissemination and decreasing the expected prevalence value for the studied agents [87]. Another variable that could influence animal contact is the existence of functional fences. Despite its importance as a reported risk factor in other studies [88,89], only six of the fourteen Enterobacteria-positive BPS maintained functional fences capable of retaining animals and preventing contact between them.

Being a user of INDAP or PRODESAL was determined as a factor that increased the risk of positivity to both agents. For the particular case of this study, those BPS owners who were users of INDAP/PRODESAL raised a greater number of animals, had a greater diversity of species and maintained several reservoir species that are included in various passive surveillance programs carried out by official entities belonging to SAG [70,90]. In addition, BPS owners who are users of INDAP or PRODESAL may belong to programs in the areas of forestry, agriculture or livestock, and therefore they do not necessarily receive veterinary advice for their animals. Furthermore, most BPS owners that were users of these entities only received economic aid. According to the above, it may be expected that INDAP/PRODESAL use represents a factor that increases the risk of positivity to Enterobacteriaceae.

## 5. Conclusions

The biosecurity measures observed empirically in BPS were factors that are not adopted as routine practices in the management of these systems. This fact could be explained by the infrequent adoption or updating of measures aimed at the handling of animals and biosecurity conditions, or by the fact that efforts were directed to other primary or secondary economic activities that provided an income to the family. Another relevant factor was the low frequency of relationships between BPS owners and professionals in the area of animal health, due to multiple causes, which generates little epidemiological surveillance of some zoonotic pathogens and their consequent under-notification.

This study represents an actualization of the epidemiological situation with regard to *S. enterica*, observing a similar behavior to that previously described for this region, and it is the first report on the circulation of STEC in these neglected animal populations. Since these bacteria are zoonotic pathogens that could have a great impact on public health, the results of this study highlight the need to generate integrated surveillance programs for BPS. These programs could be based on risk, categorizing existing risk factors to establish which of them need immediate attention. In addition, in order to be successful, surveillance programs should be carried out in conjunction with the people for whom the actions to be implemented are intended, under a One Health approach. It is necessary for the generated relationship between official health entities and BPS owners to be based on trust, in order to obtain an effective and reliable two-way line of communication. In this way, by integrating scientific tools with the empirical learning that the owners possess, it will also be possible to better understand the social and cultural context that surrounds these systems, which is an essential task for the generation of effective preventive measures to stop possible future outbreaks in high-risk populations.

## Figures and Tables

**Table 1:** Sample size determination by province, Metropolitana region.

Province	N° BPS with Birds	N° BPS with Swine	Sample Size
Melipilla	1910	202	30
Chacabuco	426	78	11
Santiago	244	61	9
Cordillera	237	29	4
Talagante	387	36	5
Maipo	632	92	13
Total			73

**Table 2:** Descriptive characterization of species kept in BPS in the Metropolitana region.

Species	N° BPS	%	AM ^1^ Animals/BPS	Min.	Max.	SD+ ^2^
Birds	40	47.06%	59.98	7	524	123.51
Chickens only	43	50.59%	57.51	3	1000	120.92
Pigs	18	21.17%	6.30	1	22	3.65
Horses	25	29.40%	3.56	1	10	2.17
Sheep	11	12.90%	8.27	1	30	4.49
Goats	5	5.80%	2.50	1	4	0.72
Cows	13	15.20%	11.61	1	40	6.62
Rabbits	4	4.70%	7.50	1	15	3.30
Dogs	60	70.60%	3.92	1	20	3.74
Cats	43	50.60%	2.58	1	8	1.90
Birds	40	47.06%	59.98	7	524	123.51

^1^ Arithmetic mean. ^2^ Standard deviation.

**Table 3:** Handling, health and biosecurity characterization of BPS from Metropolitana region.

Parameter	n	AF ^1^ Yes	RF+ ^2^ Yes	AF ^1^ No	RF+ ^2^ No
General handling characteristics					
Consumption and/or sale of animal products	85	80	0.94	5	0.06
Animal breeding at least 20 years	85	51	0.60	29	0.34
Animal handling by one person	85	59	0.69	22	0.26
Animals kept in mixed confinement (free-range during the day and confined during night)	85	58	0.68	27	0.32
Seasonal variation in the number of animals kept	85	35	0.41	50	0.59
Produce their own replacement animals	85	62	0.73	21	0.25
Handle sick animals	85	42	0.49	42	0.49
Receive veterinary assistance or diagnosis	85	15	0.18	70	0.82
Exchanging embryonated eggs	85	3	0.04	80	0.94
Debeaked chickens	85	3	0.04	79	0.93
Biosecurity characteristics					
Contact between visitors and BPS animals	85	64	0.75	20	0.24
Animals had access to a non-potable water source	85	40	0.47	45	0.53
Appropriate dead animal disposal	85	40	0.47	44	0.52
BPS neighbors (surrounded by other BPS)	85	42	0.49	40	0.47
Contact with wildlife animals	85	69	0.81	15	0.18
Contact with neighboring BPS	85	44	0.52	40	0.47
Contact between chickens and pigs	8	5	0.63	3	0.37
Functional fences	85	48	0.56	37	0.44
Footbath	85	0	0	85	1
Pre-entry disinfection	85	0	0	85	1
Post-stay disinfection	85	0	0	85	1
Watercourse inside the BPS	85	52	0.61	33	0.39
Nearby wetlands	85	1	0.01	84	0.99
Neighbors with birds/pigs	85	42	0.49	43	0.51
Proximity to intensive poultry/swine production	85	9	0.11	76	0.89
Contact between BPS animals	85	61	0.72	24	0.28
Indoor pets	85	3	0.04	82	0.96
Contact between poultry and neighboring pets	85	37	0.44	48	0.56
Pet access to animal waste	85	77	0.91	8	0.09
Contact between BPS animals and sick people	85	79	0.93	6	0.07
Relationship with government agricultural entities					
Official veterinary service visits	85	16	0.19	69	0.81
Official veterinary service sampling	85	11	0.13	74	0.87
Official veterinary service results information	85	3	0.04	82	0.96
Official veterinary service returns to BPS	85	5	0.06	80	0.94
INDAP/PRODESAL users	85	14	0.16	71	0.84

^1^ Absolute frequency. ^2^ Relative frequency.

**Table 4:** Number of positive BPS with respect to STEC and *S. enterica* by province.

Province	N° BPS	N° BPS + *S. enterica*	Province Prevalence	N° BPS + STEC	Province Prevalence
Melipilla	34	2	5.99%	4	11.76%
Talagante	7	-	-	-	-
Cordillera	5	1	20%	3	60%
Maipo	16	1	6.25%	1	6.25%
Chacabuco	13	-	-	2	15.38%
Santiago	10	-	-	-	-
Regional total	85	4	4.71%	10	11.76%

**Table 5:** Positive samples for STEC and *S. enterica* by province, BPS, animal species and Stx type.

Pathogen	Province	Code	Animal Specie	Stx1	Stx2
STEC	Melipilla	ME001	Cattle	1	1
			Cattle	1	1
			Cattle	1	1
			Swine	1	1
		ME010	Duck	0	1
			Duck	0	1
			Duck	1	0
		ME011	Hens	1	0
			Sheep	1	0
		ME024	Sheep	1	0
	Cordillera	CORD001	Cattle	0	1
		CORD003	Sheep	1	0
		CORD004	Sheep	1	0
			Goat	1	0
	Maipo	MAI009	Hens	0	1
	Chacabuco	CHAC003	Sheep	1	0
			Sheep	1	0
			Cattle	1	0
		CHAC010	Goat	1	0
			Sheep	1	0
*S. enterica*	Melipilla	ME023	Hens		
			Goose		
		ME033	Hens		
	Cordillera	CORD002	Hens		
	Maipo	MAI013	Hens		

**Table 6:** Final model results from the multivariable logistic regression analysis. The *p*-value, odds ratio (OR) and 95% confidence interval (C.I.) with lower and upper limits are reported for risk factors associated with positivity to STEC in BPS.

Variable	Categories	*p*-Value	OR	95% IC	
				Lower	Upper
(Intercept)		0.001	0.008	0	0.141
Gini–Simpson index		0.030	1.717	1.054	2.799
Functional fences	No	reference			
	Yes	0.272	0.129	0.003	5.006
Proximity to intensive poultry/swine production	No	reference			
	Yes	0.019	20.645	1.648	258.706
Official veterinary service returns to BPS	No	reference			
	Yes	0.098	17.087	0.59	495.11
Contact between poultry and neighboring pets	No	reference			
	Yes	0.219	4.41	0.415	46.88
Interaction: Functional fences/Contact between poultry and neighboring pets		0.747	0.53	0.011	24.902

**Table 7:** Final model results from the multivariable logistic regression analysis. The *p*-value, odds ratio (OR) and 95% confidence interval (C.I.) with lower and upper limits are reported for risk factors associated with positivity to *S. enterica* in BPS.

Variable	Categories	*p*-Value	OR	95% IC	
				Lower	Upper
(Intercept)		0.001	0.013	0.002	0.092
Exchanges embryonated eggs	No	reference			
	Yes	0.021	39	1.745	871.724
Presence of debeaked chickens	No	reference			
	Yes	0.001	156	6.979	3486.896

**Table 8:** Final model results from the multivariable logistic regression analysis. The *p*-value, odds ratio (OR) and 95% confidence interval (C.I.) with lower and upper limits are reported for risk factors associated with positivity to STEC and *S. enterica* in BPS.

Variable	Categories	*p*-Value	OR	95% IC	
				Lower	Upper
(Intercept)		0.079	0.172	0.024	1.229
Gini–Simpson index		0.030	1.544	1.044	2.284
Type of confinement	Free	reference			
	Mixed	0.002	0.019	0.001	2.796
	Permanent	0.403	0.466	0.078	2.796
INDAP/PRODESAL users	No	reference			
	Yes	0.023	15.02	1.465	154.082

None of the evaluated interactions showed statistical significance (*p* > 0.05).

## Data Availability

The data presented in this study are available on request from the corresponding author. The data are not publicly available because they are part of an ongoing project not yet published.

## Supplementary Materials

The following are available online at https://www.mdpi.com/article/10.3390/ijerph182010730/s1, applied questionnaire.
