# Supplementary material for: Risk Factors for Positivity to Shiga Toxin-Producing Escherichia coli and Salmonella enterica in Backyard Production Systems Animals from Metropolitana Region, Chile: A Threat to Public Health?
Source: Int J Environ Res Public Health. 2021 Oct 13;18(20):10730. doi: 10.3390/ijerph182010730 (PMC8535594; doi:10.3390/ijerph182010730)
Supplement: Supplementary file 1 [file ijerph-18-10730-s001.zip › ijerph-1384377-supplementary.pdf]

BPS_ID____________________

I. General background

0. Date: _____/_______/_____
1. BPS_ID:_________________
2. Contact name:_________________
3. Contact telephone number (to report diagnostic results):_____________________
4. Region:__________________
5. Province:________________
6. BPS objective: a. Livestock:____
b. Agricultural:____
c. Forestry:____
d. Mixed:____
e. Other:____

II. Social aspects

7. Primary activity of the holder:___________________________
8. Family group composition:_____________________________
9. Importance of animals for household economy:__________________(Scale of 1 to 5)

III. Management conditions

10. Productive species: a. Poultry:____ b. Swine:____ c. Other domestic:_____ d. Pets:____
Which?:______ Which?:_______ Which?:______

11. Poultry objective
a. Household consumption:____
b. Sale:___
c. Household consumption and sale:___
d. Keeping poultry as pets:____

Swine objective
a. Household consumption:____
b. Sale:___
c. Household consumption and sale:___
d. Keeping swine as pets:____

12. Which species and how many birds are kept at the time of the survey?

	Species	Total
12.a	Chicken	
12.b	Duck	
12.c	Turkey	
12.d	Goose	
12.e	Guineafowl	
12.f		

How many pigs are maintained at the time of the survey?

	Male	Female	Total
Adults			
Juvenile			
Breeders			
Piglets			

13. N° Hens:____
N° Roosters:____
N° Chicks:____

Loan breeding pig
Yes:____
No:____
Doesn’t apply:____

14.

Breeder movement
Enter:____
Leave:____
Doesn´t apply:____

15. Value per pig mount

a. Piglet
b. Money
c. Doesn't apply

16. Last poultry breeding

a. Actual
b. 0 to 1 year ago
c. 2 to 5 years ago
d. More than 5 years ago

Last swine breeding

a. Actual
b. 0 to 1 year ago
c. 2 to 5 years ago
d. More than 5 years ago

17. How long have you been rising: Poultry?

a. Less than 2 years:___
b. Between 2 to 10 years:__
c. Between 11 to 20 years:__
d. More than 20 years:__

Swine?

a. Less than 2 years:___
b. Between 2 to 10 years:__
c. Between 11 to 20 years:__
d. More than 20 years:__

18. Responsible for handling poultry

a. Man:___
b. Woman:___
c. Son/daughter:___
d. Family:___

Responsible for handling swine

a. Man:___
b. Woman:___
c. Son/daughter:___
d. Family:___

19. Responsible for trading of poultry

a. Man:___
b. Woman:___
c. Son/daughter:___
d. Family:___
e. Doesn't apply:___

Responsible for trading of swine

a. Man:___
b. Woman:___
c. Son/daughter:___
d. Family:___
e. Doesn't apply:___

20. What is the type of confinement for poultry?

a. Free
b. Permanent
c. Mix

What is the type of confinement for swine?

a. Free
b. Permanent
c. Mix

21. Does the number of birds vary throughout the year? When are there more?

a. Spring/summer:____
b. Autumn/Winter:____
c. Doesn't vary:____

Does the number of pigs vary throughout the year? When are there more?

a. Spring/summer:____
b. Autumn/Winter:____
c. Doesn't vary:____

22. How do you obtain bird replacements?

a. Own replacements:____
b. Buying to neighbours:____
c. Purchasing at trade fairs:____
d. Commercial farm purchasing:____
e. Obtained through state projects:____
f. Buying from intermediaries:____

Exchange of embryonated eggs?

Yes:____
No:____

How do you obtain pig replacements?

a. Own replacements:____
b. Buying to neighbours:____
c. Purchasing at trade fairs:____
d. Commercial farm purchasing:____
e. Obtained through state projects:____
f. Buying from intermediaries:____

23. OBSERVE Presence of Broiler/Layers (trimmed beak)
   a. Yes:____
   b. No:____

OBSERVE Presence of pig with the tail docked
   a. Yes:____
   b. No:____

24. What does poultry food consist of?
   a. Organic household waste:____
   b. Poultry food:____
   c. Poultry foraging:____
   d. Cereals (e,g: corn):____

What does swine food consist of?
   a. Organic household waste:____
   b. Swine food:____
   c. Swine foraging:____
   d. Cereals (e.g: flour):____

25. Where does water for poultry come from?
   a. Well:____
   b. Creek:____
   c. Potable:____
   d. Doesn't provide:___

Where does water for swine come from?
   a. Well:____
   b. Creek:____
   c. Potable:____
   d. Doesn't provide:____

26. What do you do with dead chickens?
   a. Bury:____
   b. Burn:____
   c. Trash:____
   d. Throw away from home:____
   e. Household consumption/Sale:____
   f. Nothing:____

What do you do with dead pigs?
   a. Bury:____
   b. Burn:____
   c. Trash:____
   d. Throw away from home:____
   e. Household consumption/Sale:____
   f. Nothing:____

27. Do you recognize any disease in chickens? Signs?

____
____
____

Do you recognize any disease in pigs? Signs?

____
____
____

28. What do you do if you recognize a sick chicken?

____
____
____

What do you do if you recognize a sick pig?

____
____
____

29. Do you perform any type of sanitary management or treatment of the chickens?
   a. Drugs:____
   b. Natural products:____
   c. Doesn't perform:____

Do you perform any type of sanitary management or treatment of the pigs?
a, Drugs:____
b. Natural products:____
c. Doesn't perform:____

30. Drugs administered to chickens:

____
____
____

Drugs administered to pigs:

____
____
____

31. Do you vaccinate the chickens?
   a. Yes:____
   b. No:____

Do you vaccinate the pigs?
   a. Yes:____
   b. No:____

32. Poultry receive a visit from a veterinarian?
   a. Once a year:____
   b. More than once a year:____
   c. Doesn't receive:____

Swine receives a visit from a veterinarian?
   a. Once a year:____
   b. More than once a year:____
   c. Doesn't receive:____

IV. Biosecurity

33. Is there contact between poultry and pigs?
   a. Yes:____ b. No:____ c. Doesn't apply:____

General biosecurity conditions:

34. Functional fences a. Yes:____ b. No:____
35. Presence of footbath a. Yes:____ b. No:____
36. Disinfection prior to animal handling a, Yes:____ b. No:____
37. Disinfection after animal handling a. Yes:____ b. No:____

Environmental factors:
38. Watercourse inside the BPS a. Yes:____ b. No.____
39. Neighboring wetlands or watercourses a. Yes:____. b. No.____
40. Poultry or pigs in adjacent facilities a. Yes:____ b. No.____
41. Neighboring commercial farms a. Yes:____ b. No:____
42. Poultry have access to watercourse a. Yes.____ b. No:____

43. Poultry have or may have contact with wild birds?
   a. Yes:____ b. No:____

Swine have or may have contact with wild birds?
   a. Yes.____ b. No:____

44. Poultry have or may have contact with:
Neighboring animals: a. Yes:____ b. No:____

Swine have or may have contact with:
Neighboring animals: a. Yes.____ b. No:____

45. When a new bird enters the farm:
   a. Is separated from the group for a while:____
   b. Immediately joins the group:____
   c. Doesn't apply:____

When a new pig enters the farm:
a. Is separated from the group for a while:____
b. Immediately joins the group:____
c. Doesn't apply:____

46. Can visitors have contact with the birds?
   a. Yes.____ b. No:____

Can visitors have contact with the pigs?
a. Yes.____ b. No:____

47. Do you give eggs or chickens?
   a. Yes:____ b. No:____

Did you give away pigs?
a. Yes:____ b. No:____

48. To whom?:______________________

To whom?:________________________

V. Commercial aspects

49. What products do you obtain from poultry and which is the most important?
   a. Meat:____
   b. Eggs:____
   c. Live chickens/hens:____
   d. Guano:____

Which product obtained from the pigs is the most important?
   a. Kg/animal:____
   b. Piglets:____
   c. Others:____

50. How many eggs do you collect per day?
____

51. What is the price?
Eggs:_________
Live chickens/hens:________
Meat:_________
Guano:_________

What is the price?
Meat:____________________
Piglets:___________________
Others:___________________

Other:___________

52. What is the income from poultry used for?
   a. Poultry feed:____
   b. Household expenses:____
   c. Clothes:____
   d. Children's school:____
   e. Other:_______________________

What is the income from swine used for?
   a. Swine feed:____
   b. Household expenses:____
   c. Clothes:____
   d. Children's school:____
   e. Other:_______________________________

53. If you sell, what is your main market?
   a. Neighbors/ Family:____
   b. Tourists:____
   c. Local markets:____
   d. Intermediate:____
   e. More than one market:____
   f. Restaurant:____

If you sell, what is your main market?
   a. Neighbors/Family:____
   b. Tourists:____
   c. Local markets:____
   d. Intermediate:____
   e. More than one market:____
   f. Restaurant:____

54. How many eggs do you sell per month?

____

How many pigs do you sell per year/season?

____

55. How many live chickens/hens do you sell per month?

____

How many kilograms?

____

56. How many eggs do you eat per week?

____

How many pigs do you slaughter per year?

____

57. How many chickens do you slaughter per week?

____

58. How many eggs do you buy per month (from market)?

____

59. How much poultry meat do you buy per month?

____

60. How much do you spend on poultry feeding per month?

____

How much do you spend on feeding swine per month?

____

61. Animals are inspected or visited by SAG:
   a. Yes:____ b. No:____

62. If you receive SAG visits, do they take samples?
   a. Yes:____ b. No:____

63. SAG sends the results?
   a. Yes:____ b. No:____

64. SAG returns to the BPS?
   a. Yes.____ b. No:____

65. Animals receive visits or inspection by INDAP/PRODESAL?
   a. Yes:____ b. No:____

Any of the members of the family group have presented some of the following signs and/or symptoms during the last month:

66. Diarrhea
   a. Yes:____   b. No:____

67. Fever
   a. Yes:____   b. No:____

68. Vomits
   a. Yes:____   b. No:____

69. Inappetence
   a. Yes:____   b. No:____

70. Muscle pain
   a. Yes.____   b. No:____

71. Do people with digestive disorders have the possibility to handle animals?
   a. Yes:____   b. No:____

72. Are pets indoor?
   a. Yes:____   b. No:____

73. Do pets have access to bird waste?
   a. Yes.____   b. No:____

74. Is there contact between animals kept in the BPS (between different species)?
   a. Yes:____   b. No:____

75. Is there contact between BPS's poultry and neighboring pets/animals?
   a. Yes:____   b. No:____

VI. Sample data

Type of sample birds: a. Cloacal torula:____   Type of sample pigs: a. Rectal torula:____

Type of sample from other animals: a. Rectal torula:____

Animal data

1
2
3
4
5
6
7
8
9
10
11
12
